# Evaluation of Reference Genes for Reverse Transcription Quantitative PCR Studies of Physiological Responses in the Ghost Moth, *Thitarodes armoricanus* (Lepidoptera, Hepialidae)

**DOI:** 10.1371/journal.pone.0159060

**Published:** 2016-07-08

**Authors:** Guiqing Liu, Xuehong Qiu, Li Cao, Yi Zhang, Zubing Zhan, Richou Han

**Affiliations:** Guangdong Key Laboratory of IPM in Agriculture and Public Laboratory of Wild Animal Conservation and Utilization, Guangdong Institute of Applied Biological Resources, Guangzhou, China; Laboratoire de Biologie du Développement de Villefranche-sur-Mer, FRANCE

## Abstract

Reverse transcription quantitative real-time polymerase chain reaction (RT-qPCR) is the sensitive method to quantify the expression levels of target genes on the basis of endogenous control. An appropriate reference gene set for normalization is essential for reliable results. The ghost moth, *Thitarodes armoricanus*, a host species of a medicinal fungus, *Ophiocordyceps sinensis*, is an economically important member of the Lepidoptera. Recent studies have focused on the mechanism of adaptation of this species to its high-altitude environment and host immune response to *O*. *sinensis* infection and RT-qPCR is commonly used in these studies to decipher the genetic basis of physiological functions. However, a thorough assessment of candidate reference genes in the genus *Thitarodes* is lacking. Here, the expression levels of eight candidate reference genes (ACT, EF, EIF4A, GAPDH, G6PDH, RPL13A, TUB and 18S) in *T*. *armoricanus* at different developmental stages and in different body parts of the seventh instar larvae were analyzed, along with larvae kept under low temperatures, larvae exposed to two fungal infections and larvae fed different diets. Three established software programs–Bestkeeper, geNorm and NormFinder–were employed to calculate variation among the treatments. The results revealed that the best-suited reference genes differed across the treatments, with EF, EIF4A and GAPDH found to be the best suited for the different developmental stages and larvae body parts; EF, EIF4A and RPL13A found to be the best suited for low-temperature challenge; and EF, EIF4A and TUB found to be the best suited for the fungal infections and dietary treatments. This study thus further contributes to the establishment of an accurate method for normalizing RT-qPCR results for *T*. *armoricanus* and serves as a reference for gene expression studies of related insect species.

## Introduction

Quantitative real-time reverse transcriptase polymerase chain reaction (RT-qPCR) is one of the most important techniques for quantifying mRNA expression [1]. This method has been widely used to identify genes relevant to new biological processes. For a reliable RT-qPCR assay, normalization of gene expression level using a reference gene or genes is absolutely essential to eliminate non-specific template variations between samples due to mRNA integrity, purity and reverse transcription efficiency, as well as pipetting errors [2,3]. Ideally, a reference gene would be uniformly transcribed and exhibit a similar transcription abundance to that of the target gene under different experimental conditions [4], but such an ideal reference gene remains to be identified. Growing evidences suggest that no single reference gene expression is independent from experiment conditions and some of the extensively used reference genes cannot always be employed for reliable endogenous controls for their different expression pattern in various experimental conditions [5–11]. Identification of the most stable reference gene(s) for specific experimental conditions is very important and imperative [12,13].

Several variance estimation approaches for identifying suitable reference genes for normalization have been developed using different statistical models such as the most popular softwares geNorm, NormFinder and BestKeeper [13–16]. geNorm is the first method developed to calculate the minimal number of reference genes for each experiment normalization. Similar to geNorm, NormFinder provides a stability value for each gene, but it examines the stability of each candidate reference genes independently, which is different with geNorm does. Both geNorm and NormFinder use raw data (relative quantities) as input data, the results can be easily compared. Whereas, Bestkeeper calculates the geometric mean of the best suited genes by raw Cq values of each gene and employs a pair-wise correlation analysis to determine the optimal reference genes.

Ghost moths (Lepidoptera: Hepialidae) are hosts of the fungus *Ophiocordyceps sinensis* (Berk) (syn. *Cordyceps sinensis*) [17–19]. Following infection, the fungus grows within the body cavity of the insect larva, mummifying the larva within 5–12 months and converting it into a sclerotium, from which the fruiting body of the fungus grows [20]. The parasite complex of the mummified larva and the fungal stromata forms a traditional Chinese medicine called DongChongXiaCao (DCXC), which is prized for its apparent anti-tumor [21], immunomodulating [22], hypocholesterolemic [23], hypoglycemic [24], and anti-aging [25] effects, among others.

Ghost moths inhabit alpine meadows on the Tibetan Plateau at an average altitude exceeding 4,000 m. This high-altitude environment is characterized by low mean temperatures, hypobaric hypoxia, and strong solar ultraviolet radiation [26], factors that influence the morphology, behavior, survival, reproduction, and spatial distribution of insect species [27,28]. Many entomologists regard ghost moths as a promising model for gaining a better understanding of the mechanisms of environmental adaptation [29,30]. Unlike other entomopathogenic fungi, such as *Metarhizium anisopliae* and *Beauveria bassiana*, which kill their hosts within a few days [31,32], *O*. *sinensis* requires several months of colonization before the sexual structure is produced in the mummified ghost moth [33]; as such, this fungus–insect parasite complex provides a useful model for studying the molecular interactions between entomopathogenic fungi and its insect hosts. Moreover, because of the medicinal value and limited distribution of ghost moths, it is urgent that an artificial culture system be developed for this parasite–host complex, a process that would be greatly facilitated by characterization of the physiological and biochemical processes of ghost moths.

More recent studies have used RT-qPCR technologies to identify genes involved in ghost moth cold adaptation [29,34] and immune response to fungal infection [35–37]. However, reference genes in these studies have been selected based on experience with other species rather than direct experimental evidence acquired by comprehensive study of potential reference genes. To date, improvements in transcriptomic [29,37] and genomic sequencing (unpublished work) have provided an unprecedented opportunity to enhance our understanding of the physiological and biological mechanisms of adaptation in ghost moths. We sought to identify suitable reference genes to facilitate future genomic research, focusing on the stability of eight reference genes–actin (ACT), beta-1-tubulin (TUB), 18S ribosomal RNA (18S), translation elongation factor 2 (EF), eukaryotic translation initiation factor 4A transporter-like (EIF4A), glyceraldehyde-3-phosphate dehydrogenase (GAPDH), glucose-6-phosphate 1-dehydrogenase (G6PDH) and 60S ribosomal protein L13a (RPL13A)–in *T*. *armoricanus* under different conditions, consisting of different developmental stages, larval body parts and larvae under fungi infection, low temperature and different diets. Three commonly used software packages (BestKeeper, geNorm and NormFinder) were used to identify a set of reference genes best suited to the conditions of each treatment.

## Materials and Methods

### Ethics Statement

No special permits are required for the sampling of this insect in this study. All samples were collected by the researchers with introduction letters from the Guangdong Institute of Applied Biological Resources and with the help of local herdsman.

### Insects

Pupae of *T*. *armoricanus* were collected from the slopes of Zheduo Mountain in Kangding (3,500–4,000 m above sea level), Sichuan Province, China, in June 2012. The collected pupae were housed in plastic containers at 10°C and 50–80% relative humidity until the time of adult emergence. Eggs were collected from mated females and the hatched larvae were reared on fresh carrot slices. Larvae were separated and reared individually upon reaching the second instar to avoid cannibalism. The larvae pupated after 9 larval stages. *T*. *armoricanus* completed one generation in 443–780 d, with a generation consisting of the egg stage, 9 distinct larval stages (with the occasional larva reaching a tenth instar stage, none of which survived to adulthood), the pupal stage, and finally the adult with eggs stage [19]. The experimental population was established in a laboratory in Guangzhou (43 m above sea level), Guangdong Province, China.

### Treatments

Considering the particular growth environment, specific food and its important economic value of *T*. *armoricanus* (low mean temperatures, feeding fresh root of a traditional Chinese medicine *Polygonum viviparum* L, forming a precious traditional Chinese medicine by being parasitized by *O*. *sinensis*), the stability of candidate reference genes was tested in different *T*. *armoricanus* samples. Treatments consisted of (I) different developmental stages, (II) 6 different body parts of 7^th^ instar larvae, (III) fungal infection, (IV) low temperatures and (V) different diets fed to 7^th^ instar larvae.

Developmental stages: 200 newly laid unfertilized eggs, 30 1^st^ instar larvae, 20 each of 2^nd^ to 4^th^ instar larvae, 2 each of 5^th^ to 9^th^ instar larvae, 2 female pupae, and 2 adult females and 2 adult males of *T*. *armoricanus* were collected for each replication.

Tissues: Six larvae tissues (head, thorax epidermis, abdominal epidermis, body fat, intestinal wall, and Malpighian tubules) were dissected from 3 7^th^ instar larvae using a dissection needle in 1×PBS buffer solution (phosphate buffered saline with DEPC-treated H_2_O, PBS) under a stereomicroscope (Nikon SMZ745, Japan). Each dissection was repeated in triplicate.

Fungal infection: For the fungal challenge, conidia from the *O*. *sinensis* QH1201 strain and the *Paecilomyces hepialid* PH strain-from Guangdong Institute of Applied Biological Resources were collected and diluted in sterile 1×PBS buffer solution until a concentration of 10^9^ conidia per milliliter was attained, following which 10 μL of the conidia suspension was pipetted and injected into newly molted 7^th^ instar larvae pretreated with 2 day’s starvation. The control group was injected with 10 μL of sterile 1×PBS buffer. After injection, the larvae were reared normally. Three surviving larvae without guts were collected individually on days 1, 3 and 15 post-infection for QH1201 infection and considered the control group, whereas the PH-infected group were collected at 1 d and 3 d post-infection as none of these larvae survived beyond 3 d infection.,

Low temperature: After 2 day’s starvation, 7^th^ instar larvae were exposed to temperatures of 2°C. Larvae were collected at 0 h, 3 h and 9 h post-treatment and dissected on ice immediately. Three dissected larval bodies without guts were also collected, and all testing was conducted in triplicate.

Diets: In this study, three feeding treatments including non-feeding, feeding carrot (*Daucus carota*) and feeding fresh root of *P*. *viviparum* L were divided. 30 7^th^ instar larvae pretreated with 2 day’s starvation were then fed with each diet at 10°C. Larvae were collected at 7 d and 14 d post-treatment and dissected on ice immediately. Three dissected larval bodies without guts were also collected. The guts were rinsed in DEPC-treated H_2_O three times, and guts taken from three larvae were mixed as one sample. All samples were in triplicate.

### Isolation of tissue RNA and synthesis of cDNA

All samples were preserved in RNAlater solution (Ambion, USA) and stored at -80°C. Total RNA was extracted with TRIzol reagent (Invitrogen, USA) in accordance with the manufacturer’s instructions. Extraction was followed by DNase I treatment (TransGen, China) to eliminate potential genomic DNA in all samples. The purity and quantity of RNA was measured with an Onedrop OD-1000+ spectrophotometer (Onedrop, China). The integrity of RNA was checked via 1% agarose gel electrophoresis, and only RNA samples with high integrity and for which the A260/A280 ratio ranged from 2.000 to 2.124 and the A260/A230 ratio was superior to 2.0 were used to produce the first strand cDNA. The first strand cDNA was synthesized from 1 μg of total RNA from 20 μl total volume using the PrimeScript^™^ RT Reagent Kit with gDNA Eraser (TaKaRa, Japan) in accordance with the manufacturer’s instructions. No template and no-reverse transcription control were run for each reverse transcription. After the reverse transcription, synthesized cDNA was stored at -20°C until use.

### Quantitative real-time PCR

The sequences of all candidate reference genes can be downloaded from GenBank or obtained from genomic and transcriptomic sequencing data (unpublished) and cloned. The PCR primers used for quantification of the genes encoding ACT, TUB, GAPD, G6PD, 18S, EF2, EFg, EIF4A, and RPL13 are shown in Table 1. The primers were designed with the software Primer 3 (version 0.4.0) [http://frodo.wi.mit.edu/primer3/] and Primer Premier 5.0. Primers were verified for specificity and the sizes of PCR products were checked with gel electrophoresis in the experiments. The identities of PCR products were further confirmed by sequence analysis.

**Table 1:** Details of the primers pairs used for RT-qPCR.

Gene name (abbreviation)	Accession number	Primer Sequence (5’-3’)	Amplicon length (bp)	Tm (°C)	Efficiency (%)	R2
Actin (ACT)	KU664390	F: GAGCCGTCTTTCCATCCAT	192	59.80	85.1	0.990
		R: TGTAGAAGGTGTGATGCCAGAT				
Beta-1-tubulin (TUB)	KU664388	F: GCACCCTCCTCATCTCCAAG	209	60.10	103.7	0.998
		R: GAGTTTGAGCGTGCGGAAG				
18S ribosomal (18S)	JN036435	F: CTGAGAAACGGCTACCACATC	204	59.15	101.0	0.999
		R: GCTATTGGAGCTGGAATTACC				
60S ribosomal protein L13 a (RPL13A)	KU664391	F: CATTTCAGAGCACCATCCAAG	236	59.10	105	0.993
		R: CGATACTTCCAGCCAACTTCAT				
Glyceraldehyde-3-phosphate dehydrogenase (GAPDH)	KU664394	F: TTAGTCGTCAATGGCAACAAG	207	59.02	87.2	0.990
		R: GACACCGACGACGAACATAG				
Glucose-6-phosphate 1-dehydrogenase (G6PDH)	KU664393	F: TGACACCCACTTTCGCCT	237	59.15	102.5	0.990
		R: GTCTTGCCTGGTGCTTTACA				
Translation elongation factor 2 (EF)	KU664389	F: TCGGGAGAACACATCATTGC	221	59.65	101.5	0.999
		R: GGAAGACCGTCGGGCATAG				
Translation initiation factor 4A transporter-like (EIF4A)	KU664392	F: GGAATGGAACCTGATGGAGT	188	59.20	101.9	1.000
		R: GACTGAGCCTGAGCAATGAC				

For RT-qPCR, the 25-μl reaction system consisted of 2 μl of diluted cDNA, 12.5 μl of SYBR^®^ Premix Ex Taq II (Tli RNaseH Plus) (TaKaRa, Japan) and 0.2 mM of each primer. All reactions were performed on a Stratagene MX3000P qPCR system (Stratagene, Santa Clara, CA, USA) in accordance with the manufacturer's instructions. Thermal cycling conditions were set to 95°C for 1 min of initial denaturation, followed by 40 cycles of 95°C for 15 s, 60°C for 30 s, and 72°C for 30 s of amplification. After reaction, a melting curve analysis from 55°C to 95°C was applied to all reactions to ensure consistency and specificity of the amplified product. A 5-fold dilution series of cDNA from an adult female was set as the standard for construction of a relative standard curve and determination of the PCR efficiency that would be used in converting quantification cycles (Cq values) into raw data (relative quantities).

### Expression stability analysis

BestKeeper [15], geNorm [13] and Normfinder [14] were used to evaluate gene expression stability. BestKeeper uses Cq values and PCR efficiency to determine the genes of highest reliability and combines them into an index based on the coefficient of determination and the P value [15]. The relative quantities converted from the raw Cq values (the highest relative quantity for each gene was set to 1) were used as input data for geNorm and NormFinder. geNorm calculates an expression stability value (*M*) for each reference gene; the *M* value, as the average pairwise variation *V* of one gene from all other tested reference genes, is inversely related to expression stability. Genes are ranked by a repeated process of stepwise exclusion of the genes with the highest *M* value, and values of Vn/n+1 below 0.15 indicate that no additional genes are required for normalization [13]. Similar to geNorm, NormFinder provides a stability value for each gene, which is a direct measure of the estimated expression variation that enables the users to evaluate the systematic error introduced when using the gene for normalization. This model-based approach to ranking expression stability is theoretically less sensitive to co-regulation of the candidate normalization genes than are the BestKeeper and geNorm approaches [14].

## Results

### Expression profiles of candidate reference genes

For each pair of primers, a dissociation curve after 40 cycles of amplification revealed that the primers amplified a single PCR product. All PCRs displayed a coefficient of correlation higher than 0.99. The PCR efficiency of the 8 candidate reference genes ranged from a low of 85.1% for ACT to a high of 105% for RPL13A, as shown in Table 1.

Biological samples (3 per treatment, with the exception of the different developmental stage, for which there were 2) were run in 3 technical replicates. The raw Cq values ranged from 7.37 (18S) to 25.35 (G6PDH) for the different developmental stages, from 7.53 (18S) to 25.02 (G6PDH) for the different larvae body parts, from 9.29 (18S) to 25.79 (G6PDH) under low temperatures, from 7.94 (18S) to 24.47 (G6PDH) under fungal infection and from 7.53 (18S) to 26.65 (G6PDH) among the different diets. The standard deviation (SD) of the Cq values for each gene for each treatment was consistent with SDs within 0.5 Cq (Table 2).

**Table 2:** BestKeeper analysis for candidate reference genes based on quantification cycle values (Cq).

	ACT	EF	EIF4A	GAPDH	G6PDH	RPL13A	TUB	18S
Developmental stage								
N	78	78	78	78	78	78	78	78
Geometric Mean (Cq)	19.38	18.98	19.97	20.71	23.62	18.31	18.79	8.02
Standard Deviation (±Cq)	0.39	0.83	0.99	1.00	0.77	0.97	0.73	0.23
Coefficient of Determination	0.086	0.794	0.899	0.821	0.681	0.691	0.729	0.052
P value	0.009	0.001	0.001	0.001	0.001	0.001	0.001	0.044
Body parts								
N	54	54	54	54	54	54	54	54
Geometric Mean (Cq)	19.22	17.78	18.69	19.25	23.17	17.92	18.79	8.29
Standard Deviation (±Cq)	0.49	0.40	0.82	0.79	0.67	0.33	0.99	0.40
Coefficient of Determination	0.280	0.857	0.834	0.781	0.073	0.106	0.667	0.094
P value	0.001	0.001	0.001	0.001	0.047	0.016	0.001	0.024
Low temperature								
N	27	27	27	27	27	27	27	27
Geometric Mean (Cq)	22.47	20.23	20.29	22.34	24.93	20.28	22.65	9.52
Standard Deviation (±Cq)	0.50	0.97	0.40	0.99	0.83	0.95	0.56	0.11
Coefficient of Determination	0.676	0.978	0.992	0.945	0.856	0.984	0.955	0.701
P value	0.001	0.001	0.001	0.001	0.001	0.001	0.001	0.001
Fungi infection								
N	54	54	54	54	54	54	54	54
Geometric Mean (Cq)	20.62	19.40	19.81	21.63	23.90	19.31	19.35	8.62
Standard Deviation (±Cq)	0.96	0.57	0.32	0.83	0.29	0.72	0.66	0.37
Coefficient of Determination	0.637	0.740	0.096	0.819	0.118	0.085	0.714	0.587
P value	0.001	0.001	0.023	0.001	0.011	0.032	0.001	0.001
Diets								
N	54	54	54	54	54	54	54	54
Geometric Mean (Cq)	22.90	22.71	21.89	23.11	25.57	21.27	22.93	8.15
Standard Deviation (±Cq)	0.34	0.40	0.47	0.56	0.62	0.39	0.53	0.57
Coefficient of Determination	0.731	0.616	0.489	0.371	0.438	0.362	0.706	0.282
P value	0.001	0.001	0.001	0.001	0.001	0.001	0.001	0.001

To ensure the accuracy of the estimates for expression levels, the integrity of individual samples were tested through an intrinsic variation (InVar) calculation based on BestKeeper, for which removal is recommended in cases where there is in excess of 3-fold over- or under-expression [15]. The InVar of the samples had low Cq variations (< 1), and none of the samples had an *x*-fold higher than 1.32 (S1 Table).

### Expression stability analysis

#### BestKeeper

For each biotic condition, analyses with BestKeeper revealed an overall stability in gene expression (SD < 1) for the 8 selected candidate reference genes (Table 2). These 8 genes were significantly correlated to the BestKeeper index (P < 0.001). According to the coefficient of determination of each gene, the 3 most stable genes were EIF4A, GAPDH and EF for the different developmental stages; EF, EIF4A and GAPDH for the different body parts; EIF4A, RPL13A and EF for the low-temperature challenge; GAPDH, EF and TUB for fungal infection; and ACT, TUB and EF for the different diets treatment (Table 2).

#### geNorm

**Fig 1:**
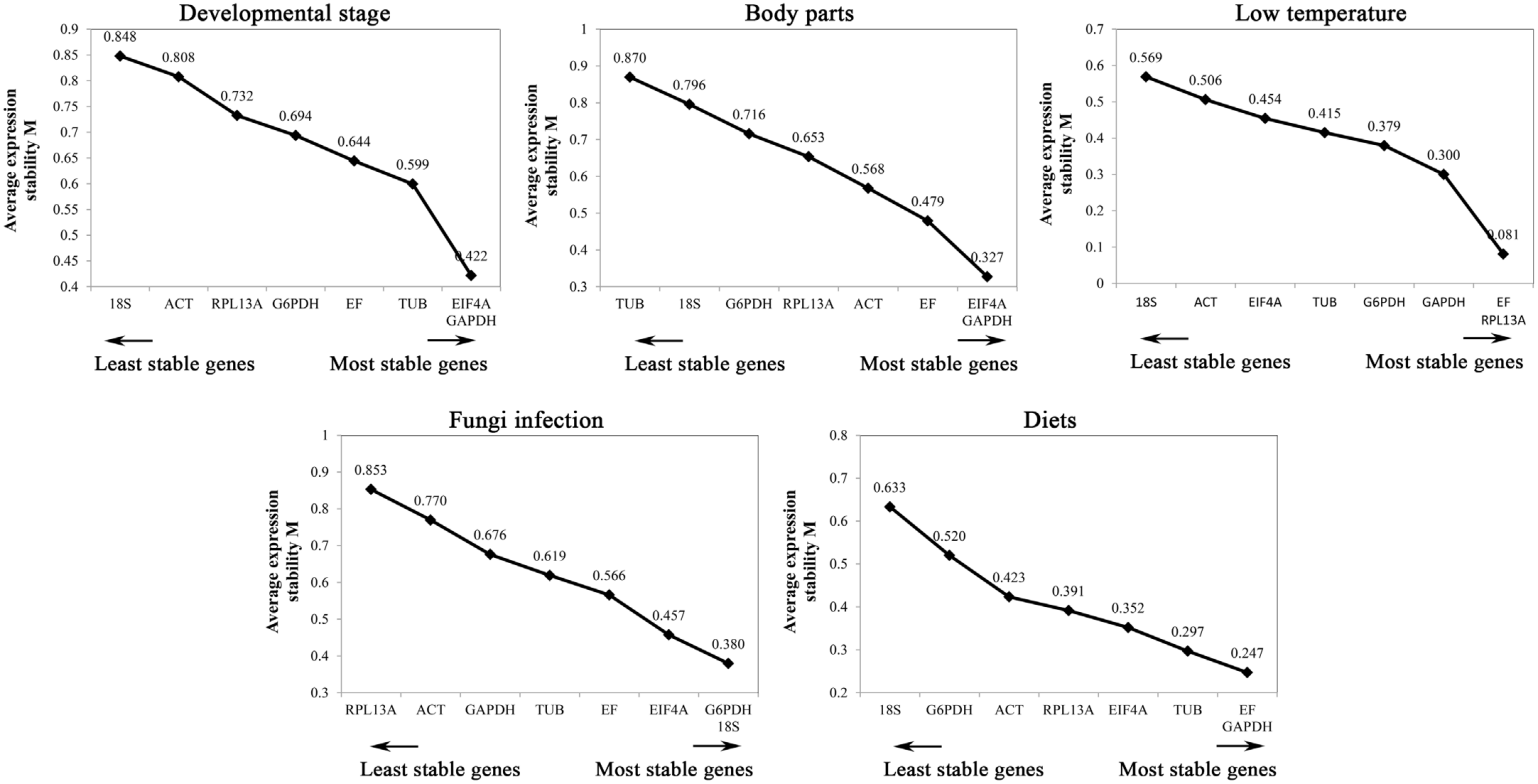
Average expression stability values of candidate reference genes in different samples determined by geNorm analysis.

Candidate reference genes were ranked from the most to the least stable under the different treatment conditions based on their *M* values obtained with the geNorm program. All *M* values were lower than 1.5, indicating that the expression levels of all of the candidate genes were relatively stable. However, the most stable or least stable reference genes differed among the treatments (Fig 1). The 3 most stable genes were EIF4A, GAPDH and TUB for the different developmental stages; EIF4A, GAPDH and EF for the different body parts; EF, RPL13A and GAPDH for the low-temperature challenge; G6PDH, 18S and EIF4A for fungal infection; and EF, GAPDH and TUB for the different diets treatment (Fig 1).

**Fig 2:**
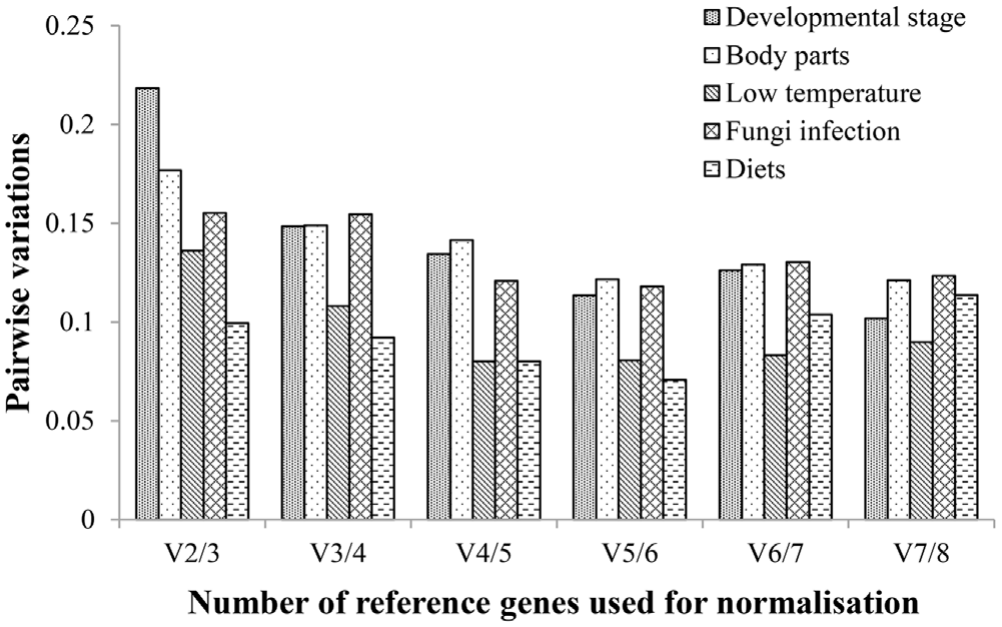
Determination of the optimal number of reference genes for an accurate normalization by geNorm. The pairwise variation (Vn/Vn+1) between two sequential normalization factors determines the optimal numbers of reference genes required for normalization. Pairwise variation values below 0.15 indicate that additional reference genes will not have a significant effect and should be excluded from the normalization.

Pairwise variation values were also calculated to determine the optimal number of reference genes for normalization, with a cut-off value of 0.15, below which the inclusion of an additional reference gene is not required [13]. The pairwise variation analyses showed that 3 reference genes for different developmental stages and body parts, and 2 reference genes for the low temperature, fungal infection and dietary treatments, might be enough to normalize the expression values of target genes (Fig 2).

#### NormFinder

**Fig 3:**
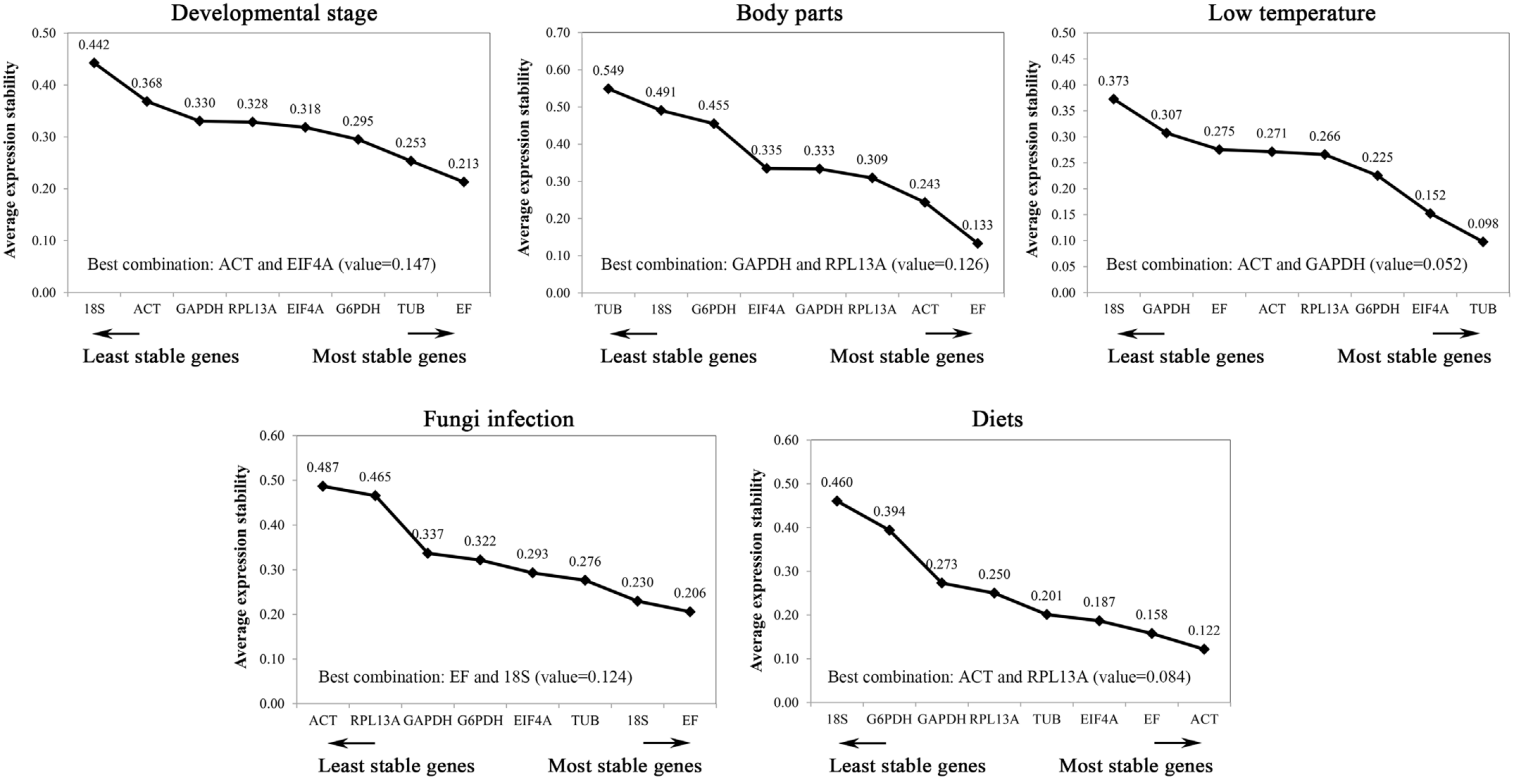
Average expression stability values of candidate reference genes in different samples determined by NormFinder analysis.

The NormFinder program was also used to identify suitable reference genes under the different treatments. This approach also demonstrated that the most stable genes differed among the various treatments: EF, TUB and G6PDH were found to be the highest ranked genes for the different developmental stages; EF, ACT and RPL13A for the different body parts; TUB, EIF4A and G6PDH for the low-temperature challenge; EF, 18S and TUB for the fungal infection; and ACT, EF and EIF4A for the dietary treatment (Fig 3). Interestingly, with the exception of the fungal infection treatment, the top combination of the two candidate reference genes to reduce the NormFinder stability value was not the combination of the two top ranked genes under the different biological conditions (Fig 3).

### Ranking of *T*. *armoricanus* reference genes with and without 18S

The rankings of the best-suited reference genes produced by the 3 programs were significantly different (Table 3). As the use of 18S for normalization is problematic given its great expression abundance, rankings were generated again after removal of 18S from the list of candidate reference genes, following which there was greater consistency among the 3 programs in the rankings of the best-suited reference genes (Table 4). With the exception of NormFinder, the same 3 genes–EF, EIF4A and GAPDH–were identified as the best-suited reference genes for the majority of the treatments, albeit in varying order (EF, EIF4A and GAPDH for the different developmental stages and body parts; EF, EIF4A and TUB for the fungal infection and dietary treatments). For the low-temperature challenge, EF, RPL13A and EIF4A were identified as the best-suited reference genes (Table 4).

**Table 3:** Ranking of candidate reference genes based on BestKeeper, geNorm and NormFinder analyses. Candidates are listed from top to bottom in order of decreasing expression stability.

Biotic conditions	Bestkeeper	geNorm	NormFinder
Developmental stage	EIF4A	EIF4A/GAPDH	EF
	GAPDH		TUB
	EF	TUB	G6PDH
	TUB	EF	EIF4A
	RPL13A	G6PDH	RPL13A
	G6PDH	RPL13A	GAPDH
	ACT	ACT	ACT
	18S	18S	18S
Body parts	EF	EIF4A/GAPDH	EF
	EIF4A		ACT
	GAPDH	EF	RPL13A
	TUB	ACT	GAPDH
	ACT	RPL13A	EIF4A
	RPL13A	G6PDH	G6PDH
	18S	18S	18S
	G6PDH	TUB	TUB
Low temperature	EIF4A	EF/ RPL13A	TUB
	RPL13A		EIF4A
	EF	GAPDH	G6PDH
	TUB	G6PDH	RPL13A
	GAPDH	TUB	ACT
	G6PDH	EIF4A	EF
	18S	ACT	GAPDH
	ACT	18S	18S
Fungi infection	GAPDH	G6PDH/18S	EF
	EF		18S
	TUB	EIF4A	TUB
	ACT	EF	EIF4A
	18S	TUB	G6PDH
	G6PDH	GAPDH	GAPDH
	EIF4A	ACT	RPL13A
	RPL13A	RPL13A	ACT
Diets	ACT	EF/GAPDH	ACT
	TUB		EF
	EF	TUB	EIF4A
	EIF4A	EIF4A	TUB
	G6PDH	RPL13A	RPL13A
	GAPDH	ACT	GAPDH
	RPL13A	G6PDH	G6PDH
	18S	18S	18S

**Table 4:** Ranking of candidate reference genes based on BestKeeper, geNorm and NormFinder analyses when 18S was removed. Candidates are listed from top to bottom in order of decreasing expression stability.

Biotic conditions	Bestkeeper	geNorm	NormFinder
Developmental stage	EF	EIF4A/GAPDH	EF
	EIF4A		TUB
	GAPDH	TUB	EIF4A
	TUB	EF	GAPDH
	G6PDH	G6PDH	RPL13A
	RPL13A	RPL13A	G6PDH
	ACT	ACT	ACT
Body parts	EIF4A	EIF4A/GAPDH	EF
	GAPDH		ACT
	EF	EF	GAPDH
	TUB	ACT	EIF4A
	ACT	RPL13A	RPL13A
	G6PDH	G6PDH	G6PDH
	RPL13A	TUB	TUB
Low temperature	EIF4A	EF/ RPL13A	TUB
	RPL13A		EIF4A
	EF	GAPDH	RPL13A
	TUB	G6PDH	G6PDH
	GAPDH	TUB	EF
	G6PDH	EIF4A	GAPDH
	ACT	ACT	ACT
Fungi infection	GAPDH	EIF4A/G6PDH	EF
	EF		TUB
	TUB	EF	EIF4A
	ACT	TUB	GAPDH
	EIF4A	GAPDH	G6PDH
	RPL13A	ACT	RPL13A
	G6PDH	RPL13A	ACT
Diets	TUB	EF/GAPDH	EF
	EF		TUB
	EIF4A	TUB	ACT/EIF4A
	GAPDH	EIF4A	
	ACT	RPL13A	GAPDH
	G6PDH	ACT	RPL13A
	RPL13A	G6PDH	G6PDH

## Discussion

RT-qPCR is extensively used to determine the mRNA expression level of target genes for understanding developmental processes in a biological system [1]. However, the lack of universally stable reference genes for RT-qPCR analyses increases the risk of misinterpretation of the results [11,38]. The importance of reference genes has attracted a great deal of attention recently, and studies on the selection of reliable reference genes has been included in quantitative expression analyses of humans [39–41], animals [38,42] and plants [43]. However, reference genes included in RT-qPCR analyses of ghost moths have often been selected based on findings for other species, such as beta-actin in *T*. *pui* [29,34–36], or the NADH dehydrogenase iron-sulfur protein 3 and ribosomal protein S3 in *T*. *xiaojinensis*, for example [30,37]. To our knowledge, our research is the first direct evaluation of the expression stability of candidate reference genes in the genus *Thitarodes* across different treatments and developmental stages using RT-qPCR.

Our assessment of 8 reference genes using 3 approaches (BestKeeper, geNorm and NormFinder) demonstrated that the evaluation of reference gene stability and primer efficiency must be conducted prior to gene-expression analyses as no universal candidate reference gene(s) can be applied to all conditions and the best-suited reference gene differs depending on the biological conditions. In this study, all reference genes vary to some extent depending on conditions, but it would seem that EF and EIF4A best fulfill the universality criteria of reference genes for *T*. *armoricanus*, with TUB also a suitable reference gene that could be used for all conditions except analyses of body parts (Table 4). These genes might be preferred candidates as normalizers in gene expression analyses. The rankings of the tested reference genes differed among the 3 programs we used (Tables 3 and 4), perhaps due to variations in the algorithm of the programs.

When compared to other reference genes, 18S rRNA (18S), which is involved in protein synthesis, was transcribed with great abundance under all conditions and varied largely across the different treatments. 18S had a low coefficient of determination (Table 2) and ranked last in stability under all conditions except that of fungal infection, for which it was ranked first by geNorm analysis (Table 3). Previous studies have regarded rRNA as an ideal reference gene, given that the regulation of rRNA synthesis is independent of mRNA level [1]. However, an increasing amount of research is revealing the limitations of using 18S as a normalizer in RT-qPCR studies [7–9,13,44]. The results of the current study also indicated that 18S is not a suitable reference gene for the normalization of gene expression analysis in *T*. *armoricanus* and that the ranking of the three reference gene evaluation programs converged to a far greater degree once 18S was excluded from the analyses (Table 4). The best reference genes for use as normalizers in our study were EF, EIF4A and GAPDH for the different developmental stages and body parts; EF, RPL13A and EIF4A for the low-temperature challenge; and EF, EIF4A and TUB for both the fungal infection and dietary treatments.

As with 18S, ACT is a commonly used reference gene that is moderately expressed in most cell types and has been highly ranked as a suitable reference gene in many insect species, including *Drosophila melanogaster* [8], *Apis mellifera* [45], *Bactrocera dorsalis* [7] and *Plutella xylostella* [46]. However, compared to the other candidate reference genes examined here, ACT expression was highly variable under the different conditions and was ranked last for the different developmental stages and low-temperature treatments and was thus considered to be unsuitable as a normalizer reference gene. This result was also in accordance with some previous studies [5,6,10].

Interestingly, we found EF and EIF4A expression to be stable under all 5 treatments examined in this study compared to the other candidate genes. However, these genes have only rarely been used as normalizers for gene expression analyses in previous studies. As has been shown, EF is primarily involved in the catalysis of the GTP-dependent ribosomal translocation step during translation elongation but has also recently been found to be the best-suited reference gene for meeting the universality criteria in *D*. *melanogaster* [8], the most stable gene for the labial gland and body fat of *Bombus lucorum* [47] and for *Nilaparvata lugens* under pesticide-stress [9]. EIF4A functions as a subunit of the initiation factor complex eIF4F, which is involved in cap recognition and is required for mRNA binding to ribosome [48]. It has recently been suggested that EIF4A is appropriate for use as a reference gene in the gut of silkworm [49] and in *Trichoplusia ni* caterpillars [50]. The combination of EF and EIF4A has also been found to be useful as reference genes to normalize gene expression in the larvae of *Phratora vitellinae* [51].

It has become clear that a single reference gene is insufficient to normalize gene expression analysis [52], and an increasing number of studies are turning to multiple reference genes for normalizing functional gene expression [53]. At the same time, an overabundance of reference genes may reduce data-normalization robustness [54]. The optimal number of reference genes across different treatments was recommended by geNorm (Fig 3). The results of the present study demonstrated that the expression stability of candidate reference genes require verification and that more than one reference gene should be used to obtain accurate RT-qPCR results.

## Conclusions

This work systematically evaluated the expression stability of 8 candidate reference genes of *T*. *armoricanus* under 5 different conditions. Stability rankings were obtained from 3 programs (BestKeeper, geNorm and NormFinder), and the optimal number of reference genes was calculated using geNorm. For accurate interpretation of the expression profiles of target genes, we concluded that EF, EIF4A and GAPDH were suitable for use as normalizers for different developmental stages and body parts of larval ghost moths, whereas EF, EIF4A and RPL13A were suitable for the low-temperature challenge and EF, EIF4A and TUB were suitable for the fungal infection and dietary treatments. Our work provides a solid basis for future study on the expression profiles of development, detoxification, cold tolerance and immunity related genes of *T*. *armoricanus* at the molecular level.

## Supporting Information

S1 Table Sample integrity analysis. The intrinsic variation (InVar) is based on candidate reference genes for 3 biological samples of each treatment and control run in triplicate, and 2 biological samples of each developmental stage run in triplicate. N: numbers of reference genes.
(DOC)
