# Supplementary material for: Evaluation of Reference Genes for Reverse Transcription Quantitative PCR Studies of Physiological Responses in the Ghost Moth, Thitarodes armoricanus (Lepidoptera, Hepialidae)
Source: PLoS One. 2016 Jul 8;11(7):e0159060. doi: 10.1371/journal.pone.0159060 (PMC4938418; doi:10.1371/journal.pone.0159060)
Supplement: S1 Table — The intrinsic variation (InVar) is based on candidate reference genes for 3 biological samples of each treatment and control run in triplicate, and 2 biological samples of each developmental stage run in triplicate. N: numbers of reference genes. (DOC) [file pone.0159060.s001.doc]

**Table S1**: Sample integrity analysis. The intrinsic variation (InVar) is based on candidate reference genes for 3 biological samples of each treatment and control run in triplicate, and 2 biological samples of each developmental stage run in triplicate. N: numbers of reference genes.

| **Developmental stage** | | | | | |
| --- | --- | --- | --- | --- | --- |
|  | **N** | **Cq** | **InVar. [± Cq]** | **InVar. [ ± % Cq]** | **InVar. [± x-fold]** |
| sample 1 | 8 | 18.25 | ±0.92 | ±4.87 | ±0.88 |
| sample 2 | 8 | 18.13 | ±0.88 | ±4.71 | ±0.78 |
| sample 3 | 8 | 18.16 | ±0.89 | ±4.6 | ±0.81 |
| sample 4 | 8 | 18.08 | ±0.91 | ±4.75 | ±0.8 |
| sample 5 | 8 | 18.14 | ±0.91 | ±4.67 | ±0.82 |
| sample 6 | 8 | 18.18 | ±0.91 | ±4.69 | ±0.84 |
| sample 7 | 8 | 18.53 | ±0.58 | ±2.65 | ±0.8 |
| sample 8 | 8 | 18.49 | ±0.54 | ±2.5 | ±0.71 |
| sample 9 | 8 | 18.57 | ±0.51 | ±2.17 | ±0.69 |
| sample 10 | 8 | 18.61 | ±0.55 | ±2.21 | ±0.79 |
| sample 11 | 8 | 18.53 | ±0.53 | ±2.35 | ±0.73 |
| sample 12 | 8 | 18.59 | ±0.53 | ±2.22 | ±0.74 |
| sample 13 | 8 | 18.25 | ±0.37 | ±2.47 | ±0.34 |
| sample 14 | 8 | 18.21 | ±0.38 | ±2.32 | ±0.35 |
| sample 15 | 8 | 18.29 | ±0.37 | ±2.48 | ±0.35 |
| sample 16 | 8 | 18.28 | ±0.33 | ±2.42 | ±0.29 |
| sample 17 | 8 | 18.22 | ±0.35 | ±2.21 | ±0.31 |
| sample 18 | 8 | 18.25 | ±0.35 | ±2.28 | ±0.32 |
| sample 19 | 8 | 18.10 | ±0.45 | ±3.05 | ±0.4 |
| sample 20 | 8 | 18.05 | ±0.41 | ±2.98 | ±0.35 |
| sample 21 | 8 | 18.11 | ±0.43 | ±3.09 | ±0.38 |
| sample 22 | 8 | 18.05 | ±0.46 | ±3.54 | ±0.38 |
| sample 23 | 8 | 18.05 | ±0.44 | ±3.25 | ±0.37 |
| sample 24 | 8 | 18.12 | ±0.43 | ±2.97 | ±0.38 |
| sample 25 | 8 | 17.35 | ±0.55 | ±2.91 | ±0.33 |
| sample 26 | 8 | 17.25 | ±0.41 | ±2.07 | ±0.2 |
| sample 27 | 8 | 17.32 | ±0.44 | ±2.23 | ±0.23 |
| sample 28 | 8 | 17.27 | ±0.4 | ±1.97 | ±0.19 |
| sample 29 | 8 | 17.27 | ±0.42 | ±2.15 | ±0.21 |
| sample 30 | 8 | 17.33 | ±0.44 | ±2.24 | ±0.23 |
| sample 31 | 8 | 17.82 | ±0.26 | ±1.85 | ±0.2 |
| sample 32 | 8 | 17.84 | ±0.27 | ±1.75 | ±0.22 |
| sample 33 | 8 | 17.88 | ±0.3 | ±1.85 | ±0.25 |
| sample 34 | 8 | 17.89 | ±0.29 | ±1.74 | ±0.25 |
| sample 35 | 8 | 17.84 | ±0.26 | ±1.68 | ±0.2 |
| sample 36 | 8 | 17.87 | ±0.29 | ±1.79 | ±0.24 |
| sample 37 | 8 | 17.42 | ±0.56 | ±3.06 | ±0.31 |
| sample 38 | 8 | 17.41 | ±0.53 | ±2.98 | ±0.28 |
| sample 39 | 8 | 17.50 | ±0.56 | ±3.11 | ±0.32 |
| sample 40 | 8 | 17.45 | ±0.58 | ±3.17 | ±0.31 |
| sample 41 | 8 | 17.43 | ±0.57 | ±3.18 | ±0.31 |
| sample 42 | 8 | 17.47 | ±0.52 | ±2.94 | ±0.3 |
| sample 43 | 8 | 16.86 | ±0.73 | ±4.28 | ±0.33 |
| sample 44 | 8 | 16.86 | ±0.72 | ±4.12 | ±0.32 |
| sample 45 | 8 | 16.91 | ±0.72 | ±4.13 | ±0.32 |
| sample 46 | 8 | 16.87 | ±0.72 | ±4.21 | ±0.32 |
| sample 47 | 8 | 16.86 | ±0.71 | ±4.05 | ±0.31 |
| sample 48 | 8 | 16.90 | ±0.69 | ±4.02 | ±0.31 |
| sample 49 | 8 | 16.63 | ±0.59 | ±2.52 | ±0.21 |
| sample 50 | 8 | 16.60 | ±0.61 | ±2.54 | ±0.21 |
| sample 51 | 8 | 16.65 | ±0.6 | ±2.49 | ±0.21 |
| sample 52 | 8 | 16.62 | ±0.62 | ±2.59 | ±0.22 |
| sample 53 | 8 | 16.70 | ±0.63 | ±2.59 | ±0.22 |
| sample 54 | 8 | 16.77 | ±0.63 | ±2.58 | ±0.23 |
| sample 55 | 8 | 17.40 | ±0.77 | ±3.93 | ±0.52 |
| sample 56 | 8 | 17.32 | ±0.73 | ±3.72 | ±0.42 |
| sample 57 | 8 | 17.37 | ±0.74 | ±3.8 | ±0.43 |
| sample 58 | 8 | 17.34 | ±0.71 | ±3.64 | ±0.43 |
| sample 59 | 8 | 17.30 | ±0.74 | ±3.78 | ±0.43 |
| sample 60 | 8 | 17.39 | ±0.71 | ±3.65 | ±0.45 |
| sample 61 | 8 | 17.37 | ±0.77 | ±4.09 | ±0.58 |
| sample 62 | 8 | 17.38 | ±0.75 | ±4.01 | ±0.56 |
| sample 63 | 8 | 17.46 | ±0.76 | ±4.05 | ±0.56 |
| sample 64 | 8 | 17.44 | ±0.75 | ±4.03 | ±0.57 |
| sample 65 | 8 | 17.39 | ±0.75 | ±3.96 | ±0.55 |
| sample 66 | 8 | 17.42 | ±0.75 | ±4 | ±0.57 |
| sample 67 | 8 | 18.55 | ±0.69 | ±3.53 | ±0.77 |
| sample 68 | 8 | 18.51 | ±0.68 | ±3.52 | ±0.73 |
| sample 69 | 8 | 18.56 | ±0.69 | ±3.54 | ±0.77 |
| sample 70 | 8 | 18.56 | ±0.7 | ±3.62 | ±0.79 |
| sample 71 | 8 | 18.50 | ±0.67 | ±3.41 | ±0.73 |
| sample 72 | 8 | 18.53 | ±0.68 | ±3.47 | ±0.76 |
| sample 73 | 8 | 18.64 | ±0.6 | ±3.77 | ±0.76 |
| sample 74 | 8 | 18.57 | ±0.59 | ±3.49 | ±0.78 |
| sample 75 | 8 | 18.63 | ±0.58 | ±3.36 | ±0.77 |
| sample 76 | 8 | 18.66 | ±0.61 | ±3.47 | ±0.84 |
| sample 77 | 8 | 18.50 | ±0.63 | ±3.92 | ±0.8 |
| sample 78 | 8 | 18.59 | ±0.57 | ±3.14 | ±0.78 |

| **Body parts** | | | | | |
| --- | --- | --- | --- | --- | --- |
|  | **N** | **Cq** | **InVar. [± Cq]** | **InVar. [ ± % Cq]** | **InVar. [± x-fold]** |
| sample 1 | 8 | 17.82 | ±0.76 | ±4.98 | ±0.89 |
| sample 2 | 8 | 17.80 | ±0.76 | ±4.91 | ±0.88 |
| sample 3 | 8 | 17.81 | ±0.77 | ±4.97 | ±0.89 |
| sample 4 | 8 | 17.48 | ±0.7 | ±3.54 | ±0.56 |
| sample 5 | 8 | 17.49 | ±0.7 | ±3.62 | ±0.56 |
| sample 6 | 8 | 17.43 | ±0.74 | ±3.9 | ±0.58 |
| sample 7 | 8 | 17.93 | ±0.67 | ±3.72 | ±0.84 |
| sample 8 | 8 | 17.37 | ±0.73 | ±4.3 | ±0.59 |
| sample 9 | 8 | 17.77 | ±0.64 | ±3.6 | ±0.71 |
| sample 10 | 8 | 17.89 | ±0.82 | ±4.44 | ±0.94 |
| sample 11 | 8 | 17.86 | ±0.78 | ±4.22 | ±0.88 |
| sample 12 | 8 | 17.86 | ±0.76 | ±4.11 | ±0.88 |
| sample 13 | 8 | 17.44 | ±0.71 | ±4.23 | ±0.74 |
| sample 14 | 8 | 17.44 | ±0.73 | ±4.32 | ±0.77 |
| sample 15 | 8 | 17.43 | ±0.75 | ±4.41 | ±0.8 |
| sample 16 | 8 | 17.84 | ±0.7 | ±3.82 | ±0.75 |
| sample 17 | 8 | 17.48 | ±0.7 | ±4.18 | ±0.73 |
| sample 18 | 8 | 17.62 | ±0.68 | ±3.89 | ±0.74 |
| sample 19 | 8 | 17.75 | ±0.92 | ±5.59 | ±1.2 |
| sample 20 | 8 | 17.77 | ±0.94 | ±5.88 | ±1.32 |
| sample 21 | 8 | 17.38 | ±0.82 | ±4.99 | ±0.78 |
| sample 22 | 8 | 17.33 | ±0.8 | ±5.14 | ±0.74 |
| sample 23 | 8 | 17.33 | ±0.83 | ±5.25 | ±0.76 |
| sample 24 | 8 | 17.37 | ±0.83 | ±5.16 | ±0.76 |
| sample 25 | 8 | 17.64 | ±0.82 | ±5 | ±0.97 |
| sample 26 | 8 | 17.61 | ±0.78 | ±4.86 | ±0.86 |
| sample 27 | 8 | 17.65 | ±0.81 | ±5.02 | ±0.95 |
| sample 28 | 8 | 16.83 | ±0.56 | ±3.71 | ±0.31 |
| sample 29 | 8 | 16.80 | ±0.51 | ±3.18 | ±0.27 |
| sample 30 | 8 | 16.78 | ±0.48 | ±2.85 | ±0.24 |
| sample 31 | 8 | 17.03 | ±0.46 | ±3.22 | ±0.27 |
| sample 32 | 8 | 16.69 | ±0.44 | ±2.73 | ±0.22 |
| sample 33 | 8 | 16.87 | ±0.46 | ±2.99 | ±0.25 |
| sample 34 | 8 | 16.90 | ±0.5 | ±3.5 | ±0.29 |
| ample 35 | 8 | 16.95 | ±0.5 | ±3.68 | ±0.3 |
| sample 36 | 8 | 16.96 | ±0.49 | ±3.73 | ±0.31 |
| sample 37 | 8 | 16.92 | ±0.49 | ±3.47 | ±0.28 |
| sample 38 | 8 | 16.89 | ±0.46 | ±3.08 | ±0.25 |
| sample 39 | 8 | 16.90 | ±0.47 | ±3.4 | ±0.28 |
| sample 40 | 8 | 16.85 | ±0.52 | ±3.39 | ±0.28 |
| sample 41 | 8 | 16.83 | ±0.5 | ±3.25 | ±0.26 |
| sample 42 | 8 | 16.82 | ±0.5 | ±3.21 | ±0.26 |
| sample 43 | 8 | 16.94 | ±0.52 | ±3.21 | ±0.31 |
| sample 44 | 8 | 16.87 | ±0.52 | ±3.05 | ±0.31 |
| sample 45 | 8 | 16.86 | ±0.51 | ±2.95 | ±0.3 |
| sample 46 | 8 | 17.01 | ±0.43 | ±2.4 | ±0.3 |
| sample 47 | 8 | 17.05 | ±0.43 | ±2.27 | ±0.3 |
| sample 48 | 8 | 16.99 | ±0.43 | ±2.51 | ±0.29 |
| sample 49 | 8 | 17.15 | ±0.37 | ±2.17 | ±0.26 |
| sample 50 | 8 | 17.24 | ±0.36 | ±1.84 | ±0.25 |
| sample 51 | 8 | 17.31 | ±0.36 | ±2.24 | ±0.26 |
| sample 52 | 8 | 16.98 | ±0.41 | ±2.43 | ±0.27 |
| sample 53 | 8 | 17.20 | ±0.4 | ±2.07 | ±0.29 |
| sample 54 | 8 | 17.13 | ±0.37 | ±1.92 | ±0.26 |

| **Low temperature** | | | | | |
| --- | --- | --- | --- | --- | --- |
|  | **N** | **Cq** | **InVar. [± Cq]** | **InVar. [ ± % Cq]** | **InVar. [± x-fold]** |
| sample 1 | 8 | 19.73 | ±0.33 | ±1.48 | ±0.25 |
| sample 2 | 8 | 19.69 | ±0.33 | ±1.46 | ±0.25 |
| sample 3 | 8 | 19.73 | ±0.32 | ±1.42 | ±0.25 |
| sample 4 | 8 | 19.63 | ±0.27 | ±1.2 | ±0.19 |
| sample 5 | 8 | 19.59 | ±0.28 | ±1.29 | ±0.19 |
| sample 6 | 8 | 19.60 | ±0.27 | ±1.24 | ±0.19 |
| sample 7 | 8 | 19.68 | ±0.3 | ±1.3 | ±0.22 |
| sample 8 | 8 | 19.65 | ±0.31 | ±1.39 | ±0.22 |
| sample 9 | 8 | 19.66 | ±0.31 | ±1.35 | ±0.22 |
| sample 10 | 8 | 20.54 | ±0.47 | ±2.14 | ±0.63 |
| sample 11 | 8 | 20.48 | ±0.47 | ±2.28 | ±0.62 |
| sample 12 | 8 | 20.49 | ±0.47 | ±2.19 | ±0.61 |
| sample 13 | 8 | 20.53 | ±0.44 | ±1.99 | ±0.58 |
| sample 14 | 8 | 20.57 | ±0.45 | ±1.99 | ±0.61 |
| sample 15 | 8 | 20.60 | ±0.44 | ±1.94 | ±0.61 |
| sample 16 | 8 | 20.51 | ±0.47 | ±2.15 | ±0.63 |
| sample 17 | 8 | 20.50 | ±0.44 | ±2.01 | ±0.57 |
| sample 18 | 8 | 20.46 | ±0.46 | ±2.25 | ±0.6 |
| sample 19 | 8 | 18.84 | ±0.5 | ±2.2 | ±0.21 |
| sample 20 | 8 | 18.86 | ±0.5 | ±2.25 | ±0.22 |
| sample 21 | 8 | 18.84 | ±0.51 | ±2.26 | ±0.22 |
| sample 22 | 8 | 18.88 | ±0.49 | ±2.05 | ±0.2 |
| sample 23 | 8 | 18.89 | ±0.47 | ±1.92 | ±0.19 |
| sample 24 | 8 | 18.84 | ±0.47 | ±1.93 | ±0.19 |
| sample 25 | 8 | 18.91 | ±0.5 | ±2.22 | ±0.21 |
| sample 26 | 8 | 18.93 | ±0.51 | ±2.26 | ±0.22 |
| sample 27 | 8 | 18.96 | ±0.51 | ±2.29 | ±0.22 |

| **Fungi infection** | | | | | |
| --- | --- | --- | --- | --- | --- |
|  | **N** | **Cq** | **InVar. [± CP]** | **InVar. [ ± % CP]** | **InVar. [± x-fold]** |
| sample 1 | 8 | 17.72 | ±0.87 | ±4.41 | ±0.6 |
| sample 2 | 8 | 17.72 | ±0.87 | ±4.42 | ±0.59 |
| sample 3 | 8 | 17.79 | ±0.86 | ±4.35 | ±0.58 |
| sample 4 | 8 | 17.71 | ±0.87 | ±4.45 | ±0.6 |
| sample 5 | 8 | 17.72 | ±0.85 | ±4.32 | ±0.57 |
| sample 6 | 8 | 17.79 | ±0.85 | ±4.32 | ±0.58 |
| sample 7 | 8 | 17.82 | ±0.85 | ±4.41 | ±0.59 |
| sample 8 | 8 | 17.72 | ±0.86 | ±4.34 | ±0.57 |
| sample 9 | 8 | 17.79 | ±0.85 | ±4.32 | ±0.58 |
| sample 10 | 8 | 18.78 | ±0.27 | ±2.37 | ±0.23 |
| sample 11 | 8 | 18.71 | ±0.27 | ±2.34 | ±0.22 |
| sample 12 | 8 | 18.74 | ±0.27 | ±2.39 | ±0.22 |
| sample 13 | 8 | 18.73 | ±0.26 | ±2 | ±0.22 |
| sample 14 | 8 | 18.70 | ±0.26 | ±1.99 | ±0.21 |
| sample 15 | 8 | 18.77 | ±0.28 | ±2.35 | ±0.24 |
| sample 16 | 8 | 18.67 | ±0.26 | ±2.24 | ±0.2 |
| sample 17 | 8 | 18.72 | ±0.26 | ±2.19 | ±0.22 |
| sample 18 | 8 | 18.76 | ±0.27 | ±2.23 | ±0.23 |
| sample 19 | 8 | 18.25 | ±0.45 | ±2.19 | ±0.27 |
| sample 20 | 8 | 18.35 | ±0.52 | ±2.62 | ±0.32 |
| sample 21 | 8 | 18.28 | ±0.43 | ±2.1 | ±0.26 |
| sample 22 | 8 | 18.29 | ±0.39 | ±2.06 | ±0.24 |
| sample 23 | 8 | 18.24 | ±0.46 | ±2.43 | ±0.27 |
| sample 24 | 8 | 18.29 | ±0.4 | ±2.06 | ±0.24 |
| sample 25 | 8 | 18.24 | ±0.49 | ±2.24 | ±0.3 |
| sample 26 | 8 | 18.28 | ±0.52 | ±2.55 | ±0.32 |
| sample 27 | 8 | 18.38 | ±0.51 | ±2.85 | ±0.33 |
| sample 28 | 8 | 17.98 | ±0.6 | ±3.56 | ±0.34 |
| sample 29 | 8 | 18.00 | ±0.57 | ±3.35 | ±0.31 |
| sample 30 | 8 | 17.97 | ±0.59 | ±3.52 | ±0.33 |
| sample 31 | 8 | 17.88 | ±0.59 | ±3.63 | ±0.32 |
| sample 32 | 8 | 17.92 | ±0.59 | ±3.5 | ±0.32 |
| sample 33 | 8 | 17.89 | ±0.6 | ±3.65 | ±0.33 |
| sample 34 | 8 | 17.92 | ±0.6 | ±3.5 | ±0.34 |
| sample 35 | 8 | 17.92 | ±0.58 | ±3.32 | ±0.32 |
| sample 36 | 8 | 17.96 | ±0.6 | ±3.49 | ±0.35 |
| sample 37 | 8 | 18.55 | ±0.74 | ±3.65 | ±0.84 |
| sample 38 | 8 | 18.53 | ±0.72 | ±3.56 | ±0.82 |
| sample 39 | 8 | 18.56 | ±0.74 | ±3.7 | ±0.84 |
| sample 40 | 8 | 18.56 | ±0.69 | ±3.5 | ±0.76 |
| sample 41 | 8 | 18.58 | ±0.72 | ±3.77 | ±0.77 |
| sample 42 | 8 | 18.62 | ±0.7 | ±3.7 | ±0.78 |
| sample 43 | 8 | 18.55 | ±0.74 | ±3.63 | ±0.86 |
| sample 44 | 8 | 18.46 | ±0.7 | ±3.46 | ±0.76 |
| sample 45 | 8 | 18.62 | ±0.68 | ±3.41 | ±0.77 |
| sample 46 | 8 | 19.27 | ±0.69 | ±3.16 | ±1 |
| sample 47 | 8 | 19.24 | ±0.66 | ±3 | ±0.97 |
| sample 48 | 8 | 19.18 | ±0.73 | ±3.42 | ±1 |
| sample 49 | 8 | 19.26 | ±0.66 | ±3.01 | ±0.93 |
| sample 50 | 8 | 19.18 | ±0.73 | ±3.4 | ±1 |
| sample 51 | 8 | 19.25 | ±0.65 | ±2.97 | ±0.9 |
| sample 52 | 8 | 19.30 | ±0.66 | ±3 | ±0.95 |
| sample 53 | 8 | 19.21 | ±0.64 | ±2.93 | ±0.9 |
| sample 54 | 8 | 19.25 | ±0.65 | ±2.92 | ±0.94 |
|  |  |  |  |  |  |
| **Diets** | | | | | |
|  | **N** | **Cq** | **InVar. [± Cq]** | **InVar. [ ± % Cq]** | **InVar. [± x-fold]** |
| sample 1 | 8 | 20.38 | ±0.48 | ±2.61 | ±0.49 |
| sample 2 | 8 | 20.32 | ±0.49 | ±2.8 | ±0.48 |
| sample 3 | 8 | 20.38 | ±0.48 | ±2.61 | ±0.49 |
| sample 4 | 8 | 20.29 | ±0.39 | ±2.28 | ±0.33 |
| sample 5 | 8 | 20.24 | ±0.38 | ±2.32 | ±0.32 |
| sample 6 | 8 | 20.29 | ±0.39 | ±2.28 | ±0.33 |
| sample 7 | 8 | 20.29 | ±0.4 | ±2.31 | ±0.34 |
| sample 8 | 8 | 20.30 | ±0.39 | ±2.17 | ±0.34 |
| sample 9 | 8 | 20.24 | ±0.39 | ±2.39 | ±0.33 |
| sample 10 | 8 | 20.37 | ±0.5 | ±2.82 | ±0.42 |
| sample 11 | 8 | 20.31 | ±0.49 | ±2.84 | ±0.41 |
| sample 12 | 8 | 20.37 | ±0.5 | ±2.82 | ±0.42 |
| sample 13 | 8 | 20.44 | ±0.47 | ±2.54 | ±0.42 |
| sample 14 | 8 | 20.40 | ±0.44 | ±2.36 | ±0.38 |
| sample 15 | 8 | 20.47 | ±0.45 | ±2.26 | ±0.41 |
| sample 16 | 8 | 20.44 | ±0.47 | ±2.54 | ±0.42 |
| sample 17 | 8 | 20.40 | ±0.47 | ±2.55 | ±0.4 |
| sample 18 | 8 | 20.43 | ±0.47 | ±2.51 | ±0.41 |
| sample 19 | 8 | 20.52 | ±0.72 | ±8.05 | ±0.91 |
| sample 20 | 8 | 20.47 | ±0.72 | ±7.95 | ±0.89 |
| sample 21 | 8 | 20.51 | ±0.71 | ±7.92 | ±0.88 |
| sample 22 | 8 | 20.53 | ±0.71 | ±8.22 | ±0.95 |
| sample 23 | 8 | 20.44 | ±0.67 | ±7.48 | ±0.8 |
| sample 24 | 8 | 20.47 | ±0.64 | ±7.32 | ±0.78 |
| sample 25 | 8 | 20.53 | ±0.68 | ±7.72 | ±0.85 |
| sample 26 | 8 | 20.46 | ±0.68 | ±7.56 | ±0.82 |
| sample 27 | 8 | 20.53 | ±0.68 | ±7.67 | ±0.84 |
| sample 28 | 8 | 19.87 | ±0.44 | ±2.52 | ±0.29 |
| sample 29 | 8 | 19.85 | ±0.41 | ±2.43 | ±0.27 |
| sample 30 | 8 | 19.92 | ±0.4 | ±2.16 | ±0.28 |
| sample 31 | 8 | 19.95 | ±0.52 | ±2.52 | ±0.43 |
| sample 32 | 8 | 19.91 | ±0.51 | ±2.51 | ±0.41 |
| sample 33 | 8 | 19.97 | ±0.53 | ±2.49 | ±0.45 |
| sample 34 | 8 | 19.95 | ±0.52 | ±2.52 | ±0.43 |
| sample 35 | 8 | 19.94 | ±0.5 | ±2.46 | ±0.4 |
| sample 36 | 8 | 19.95 | ±0.5 | ±2.41 | ±0.41 |
| sample 37 | 8 | 19.20 | ±0.38 | ±1.67 | ±0.14 |
| sample 38 | 8 | 19.16 | ±0.38 | ±1.8 | ±0.14 |
| sample 39 | 8 | 19.27 | ±0.37 | ±1.45 | ±0.14 |
| sample 40 | 8 | 19.18 | ±0.43 | ±1.82 | ±0.15 |
| sample 41 | 8 | 19.23 | ±0.43 | ±1.69 | ±0.16 |
| sample 42 | 8 | 19.20 | ±0.43 | ±1.72 | ±0.15 |
| sample 43 | 8 | 19.17 | ±0.38 | ±1.81 | ±0.13 |
| sample 44 | 8 | 19.21 | ±0.39 | ±1.76 | ±0.14 |
| sample 45 | 8 | 19.17 | ±0.38 | ±1.8 | ±0.13 |
| sample 46 | 8 | 20.33 | ±0.36 | ±1.63 | ±0.37 |
| sample 47 | 8 | 20.27 | ±0.34 | ±1.42 | ±0.33 |
| sample 48 | 8 | 20.27 | ±0.34 | ±1.44 | ±0.34 |
| sample 49 | 8 | 20.41 | ±0.31 | ±1.32 | ±0.31 |
| sample 50 | 8 | 20.38 | ±0.32 | ±1.36 | ±0.3 |
| sample 51 | 8 | 20.40 | ±0.33 | ±1.35 | ±0.32 |
| sample 52 | 8 | 20.38 | ±0.32 | ±1.37 | ±0.32 |
| sample 53 | 8 | 20.40 | ±0.34 | ±1.43 | ±0.35 |
| sample 54 | 8 | 20.34 | ±0.33 | ±1.49 | ±0.32 |
